# Antibacterial effect of acidic ionized water on horse wounds bacterial isolates

**DOI:** 10.14202/vetworld.2021.1128-1132

**Published:** 2021-05-10

**Authors:** Afiqah Zafirah Abdul Rahman¹, Noraniza Mohd Adzahan, Zunita Zakaria, Abubakar Musa Mayaki

**Affiliations:** 1 Department of Farm and Exotic Animal Medicine and Surgery, Faculty of Veterinary Medicine, Universiti Putra Malaysia, 43400 UPM Serdang, Selangor, Malaysia; 2 Department of Veterinary Pathology and Microbiology, Faculty of Veterinary Medicine, Universiti Putra Malaysia, 43400 UPM Serdang, Selangor, Malaysia; 3 Department of Veterinary Medicine, Faculty of Veterinary Medicine, Usmanu Danfodiyo University, Sokoto, PMB2346, City Campus, Sokoto, Nigeria

**Keywords:** bacterial growth, ionized water, horse, wounds

## Abstract

**Background and Aim:**

Horse wounds can be easily infected with bacteria depending on the nature of its cause such as laceration, abrasion, or puncture as well as the nature of its environment. Various treatments are available in managing open wounds, including the usage of topical antibiotics and antiseptics. However, antibiotic resistance has been a major concern attributed with chronic wound infection. The aim of this study was to test the efficacy of ionized water at different pH against the growth of common bacteria from horse wounds.

**Materials and Methods:**

Ten swab samples from equine infected wounds were collected and bacteria isolation and identification were performed. The antibacterial effect of the ionized water of pH 2.5, 4.5, 7.0, and 11.5 was tested on *Staphylococcus aureus*, *Staphylococcus pseudintermedius*, *Staphylococcus intermedius*, *Escherichia coli*, *Pantoea agglomerans*, and *Klebsiella pneumoniae*. The time-kill profiles of the ionized waters were determined at time 0, 2, 4, 6, and 8 h.

**Results:**

Ionized water of pH 2.5 and 4.5 showed antibacterial activity against *S. aureus*, *S. pseudintermedius*, and *S. intermedius* with significant (p>0.05) reduction in colony-forming unit/mL within 2-8 h. The degree of bactericidal effect of the acidic ionized water differs between the species with *S. intermedius* more susceptible. However, there was no antibacterial effect at pH 2.5, 4.5, 7.0, and 11.5 on the Gram-negative bacteria tested.

**Conclusion:**

Ionized water of pH 2.5 and 4.5 is effective in minimizing the growth of Gram-positive bacteria; thus it could be of clinical importance as an antiseptic for surface wound lavage in horses.

## Introduction

Wounds mostly occur due to trauma resulting in the breakage of integrity of dermal layer, thus causing it to be exposed to external environment. Wounds in horses have the tendency to get infected due to various environmental factors such as fecal contamination, dirt, and plant debris, as well as foreign bodies [1,2]. Due to the risk of infection, the main purpose of wound management is to reduce presence of bacteria on the healing tissue. To achieve this, wound lavage, debridement, dressing, and bandaging are the methods employed in wound management apart from the use of antibiotic therapy [2]. However, the existence of antimicrobial resistance, particularly multidrug resistance bacteria, has made the use of antibiotics for wound management challenging [3]. Furthermore, most of the disinfectant or antiseptic solutions used are chemical based with varying degrees of toxicity such as skin irritation, allergic reaction, and occupational asthma in human [4].

The antibacterial effect of electrolyzed and ionized waters of acidic pH on surface microbial growth reported by previous studies suggests that these substances can be used as an antiseptic or disinfectant. More importantly, healing of cutaneous open wound and control of burn wound infection in rats have been facilitated by ionized and electrolyzed oxidized water, respectively [5,6]. The antibacterial effect of electrolyzed water has been mostly reported on raw food products in food industries as well as animal transport vehicles [7-9].

There is insufficient research on ionized water in veterinary practice. It is our belief that ionized water depending on the pH may serve as wound surface lavage solution that can control bacterial infection that is associated with chronic non-healing wound in horses. We, therefore, aim in this study to investigate the effect of ionized water at different pH at different time against the growth of bacterial isolate from horse wounds.

## Materials and Methods

### Ethical approval

All procedures were performed according to the guideline approved by the Institutional Animal Care and Use Committee, Universiti Putra Malaysia (UPM/IACUC/AUP-U073/2018).

### Study period and location

This study was conducted from August to September 2018. All the horses included in this study were cases presented at the University Veterinary Hospital (UVH) Faculty of Veterinary Medicine, Universiti Putra Malaysia (UPM). The bacteriological procedures were performed at the Bacteriology Laboratory, Faculty of Veterinary Medicine, UPM.

### Sample collection

Wound swabs were collected from 10 horses with different types of wound lesion. The wound was first rinsed with sterile saline and the swabbing was done with a sterile swab using Levine’s technique of wound sampling. The swabs were then placed in a Cary Blair transport medium and transported on ice.

### Isolation and identification of bacteria

Each individual swab sample were streaked onto blood and MacConkey agars and incubated at 37°C for 24 h. The individual bacterial colony from each agar plate was subcultured on blood and MacConkey agar at 37°C for 24 h to obtain a pure culture. Biochemical tests were conducted on the pure culture to identify the bacterial species.

### Preparation of ionized water

Ionized water was prepared using a conventional water ionizer. The ionizer setting was manipulated to produce ionized water of pH 2.4, 4.5, 7.0, and 11.5.

### Time-kill assay

The top three isolated Gram-positive (*Staphylococcus aureus*, *Staphylococcus pseudintermedius*, and *Staphylococcus intermedius*) and Gram-negative bacteria (*Escherichia coli*, *Pantoea agglomerans*, and *Klebsiella pneumoniae*) were selected to assess the time-kill effect of the ionized waters of different pH tested. The respective bacteria colonies were mixed with sterile distilled water and standardized using McFarland 0.5 to obtain a colony-forming unit (CFU) of 1.5×10^8^. The bacteria suspension (1.0 mL) was then added into respective test tube containing ionized water at different pH. Sterile distilled water was used as control. The suspensions were allowed to settle for 0, 2, 4, 6, and 8 h, and for every time interval, 0.1 mL of the bacteria suspension was taken from respective test tubes and plated onto nutrient agar which was incubated at 37°C for 24 h. All cultural analyses for each respective time were done in triplicates. The time-kill analysis to assess the bactericidal effect of the ionized water was determined by manually counting the bacteria colony growth in each agar for the time 0, 2, 4, 6, and 8 h and the CFU/mL calculated. The bactericidal effect of the ionized water is considered when the decrease in CFU/mL of the bacteria growth in relation to the initial culture inoculum was >3log10, indicating 99.9% killing of the inoculum.

### Statistical analysis

The bacterial isolates were presented using descriptive statistics. The average of the CFU/mL of the triplicate cultural analysis for each time interval was calculated and used for the time-kill curve. The difference in log CFU/mL between the control and the different pH ionized water at times 0, 2, 4, 6, and 8 h were compared using Student’s t-test. Statistical analyses were performed using GraphPad Prism version 8 (GraphPad Software, California, USA), with p<0.05 considered as statistically significant.

## Results

### Bacteria isolation

The cultural isolation of bacteria from the horse wound swab samples yielded 49 isolates comprising 20 bacterial species with 51% being Gram-negative and 49% being Gram-positive species (Table-1). The prominent species are *S. aureus* (26.53%), *E. coli* (16.33%), and *P. agglomerans* (14.29%).

**Table-1:** Bacterial species isolated from equine wound swab samples.

Class of bacteria	Bacterial species	Number of isolates	% isolates
Gram-positive bacteria	*Staphylococcus aureus*	13	26.53
	*Staphylococcus pseudintermedius*	3	6.12
	*Staphylococcus intermedius*	2	4.08
	*Streptococcus equi* ssp*. zooepidemicus*	1	2.04
	*Streptococcus iniae*	1	2.04
	*Streptococcus equi*	1	2.04
	*Streptococcus equisimilis*	1	2.04
	*Listeria monocytogenes*	1	2.04
	*Bacillus cereus*	1	2.04
Gram-negative bacteria	*Escherichia coli*	8	16.33
	*Pantoea agglomerans*	7	14.29
	*Klebsiella pneumoniae*	2	4.08
	*Plesiomonas shigelloides*	1	2.04
	*Proteus mirabilis*	1	2.04
	*Yersinia intermedia*	1	2.04
	*Yersinia frederiksenii*	1	2.04
	*Pseudomonas aeruginosa*	1	2.04
	*Klebsiella* sp*.*	1	2.04
	*Enterobacter cloacae*	1	2.04
	*Vibrio parahaemolyticus*	1	2.04

### Time-kill assay

The prominent Gram-positive: *S. aureus*, *S. pseudintermedius*, and *S. intermedius*, and Gram-negative: *E. coli*, *P. agglomerans*, and *K. pneumoniae* bacterial isolates were used for the assessment of antibacterial effect of acidic and alkaline ionized water.

**Figure-1:**
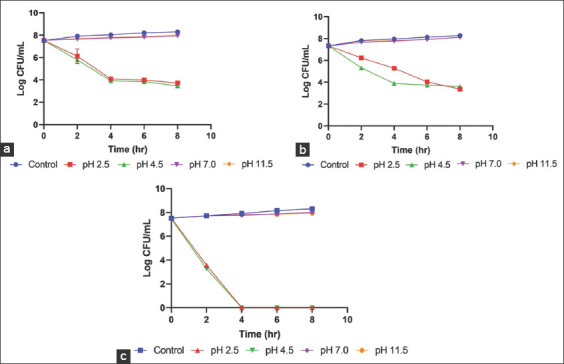
Antibacterial effect of different pH ionized waters on prominent Gram-positive bacterial isolated from horses wound. (a) *Staphylococcus aureus*, (b) *Staphylococcus pseudintermedius*, (c) *Staphylococcus intermedius*.

The time-kill assay on Gram-positive bacteria showed that ionized water at pH 7 and 11.5 were ineffective against the three bacterial species with no significant reduction in CFU/mL observed when compared to the control over the 8 h period. However, ionized water at pH 2.5 and 4.5 showed antibacterial activity against *S. aureus*, *S. pseudintermedius*, and *S. intermedius* with significant (p>0.05) reduction in CFU/mL from 2 to 8 h when compared to the control (Figure-1a-c). There was no significant difference in the antibacterial activity at pH 2.5 and 4.5 against *S. aureus*, *S. pseudintermedius*, and *S. intermedius*. However, the reduction of the bacteria cells was higher at pH 4.5 than at pH 2.5.

When comparing the antibacterial susceptibility of the three organisms to ionized water at pH 2.5 and 4.5, a bactericidal effect of pH 2.5 and 4.5 with 99.99% kill of *S. aureus* inoculum was observed between 4 and 8 h post-inoculation (Figure-1a). For *S. pseudintermedius*, bactericidal effect was observed at pH 2.5 and 4.5 between 6 and 8 h, and 4 and 8 h, respectively (Figure-1b). However, *S. intermedius* was more susceptible with 99.99% kill of the bacteria observed during the 2^nd^ h and no bacteria growth seen over the remaining 6 h (Figure-1c).

**Figure-2:**
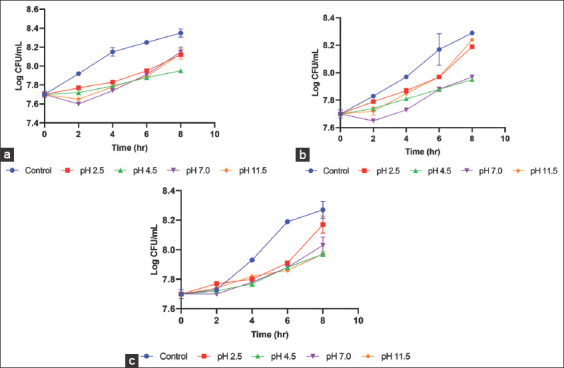
Time-kill measurements of ionized waters of different pH on Gram-negative bacterial isolated from horses wound. (a) *Escherichia coli*, (b) *Pantoea agglomerans*, and (c) *Klebsiella pneumoniae*.

Contrastingly, all the ionized water at different pH tested on *E. coli*, *P. agglomerans*, and *K. pneumoniae* showed ineffective antibacterial activities (Figure-2). Although there was slight reduction in CFU/mL of each bacterial species compared to the control, the highest reduction (50-59%) relative to the initial inoculum of *E. coli*, *P. agglomerans*, and *K. pneumoniae* was <3log 10, indicating that the ionized water at pH 2.5, 4.5, 7, and 11.5 has no bactericidal effect on the bacteria growth inhibition.

## Discussion

Wounds in horses are highly contaminated with microbial pathogens. The bacterial isolates from the wound samples are similar to earlier studies with *Staphylococcus* been the most genus identified, followed by *E. coli* [1,10]. Since the colonization of these organisms, particularly *Staphylococcus* spp., could lead to chronic wound infection, the antibacterial effect of ionized water at different pH was tested on some selected species as well as Gram-negative bacterial species. Although, studies have shown that electrolyzed water possesses an effective antibacterial activity against common surface bacterial isolate like *S. aureus* [11,12], this is the first report on the effects of ionized water on bacterial isolates of equine wounds.

In the present study, significant bactericidal activity of ionized water pH 2.5 and 4.5 was demonstrated on *S. aureus*, *S. pseudintermedius*, and *S. intermedius* using time-kill measurements. *S. aureus* decreased by 99% in the initial 2 h, and within 4-8 h, bactericidal effect with 99.99% kill was observed. Similarly, the percentage log reduction of *S. pseudintermedius* due to pH 4.5 was 99% at the 2 h and 99.99% from 6 to 8 h. In the case of ionized water pH 2.5 on *S. pseudintermedius*, the percentage reduction was 99% at the 4 h post-inoculation and 99.99% within 6-8 h. However, the susceptibility of *S. intermedius* to the acidic pH was much higher with 99.99% killing of the bacteria observed in the first 2 h and complete bacteria growth inhibition was seen over the remaining 6 h. Unlike acidic electrolyzed water which its bactericidal effect is attributed to hypochlorous acid, the antibacterial effect of acidic ionized water could be as a result of reactive oxygen species due to free radicals produce at anode chamber during ionization reaction. Contrary to the acidic ionized water, pH 7.0 and 11.5 caused a relatively low reduction with log difference relative to the initial bacteria inoculum <3log 10, indicating no bactericidal effect on the tested *Staphylococcus* species at both pH. The highest percentage reduction observed for pH 7 and 11.5 on *S. aureus* was 58.3% and 57.3% within the 8 h and 6 h, respectively. Similarly, 51.0% and 55.3% reduction in *S. intermedius* was seen within 6-8 h, while much lower reduction of 32% was observed for *S*. *pseudintermedius* at the end of 8 h. The difference in the effect of ionized water among *Staphylococcus* species could be due to genetic variants and cellular properties that the subspecies possesses. The ability to change membrane composition through the alteration of the membrane fatty acid sequence in response to the decrease or increase in environmental pH has been acknowledged [13]. Furthermore, some pathogenic bacteria also possess adaptive acid tolerance response, like the ability of the bacteria to change protein expression when exposed to acidic environment [14,15]. The significant higher effect at pH 4.5 than the pH 2.5 could be attributed to the fact that weak acids have higher antimicrobial activity due to its dissociated form that can freely pass through the cell membrane [13] and it ability to promote undesirable redox reaction which is responsible for the bactericidal effect.

For the selected Gram-negative bacteria, the time-kill measurements showed ineffective antibacterial activities of the acidic, neutral, and alkaline ionized water on *E. coli*, *P. agglomerans*, and *K. pneumoniae*. Throughout the 8 h period of the test, the log reduction difference in relative to the initial bacteria inoculum for all the pH tested was <3log 10, indicating ionized water has no bactericidal effect on the growth inhibition of Gram-negative bacteria. The highest percentage reduction for *E. coli* was observed with pH 2.5 (50%) and pH 4.5 (58%). The highest percentage reduction for *P. agglomerans* (26%-27%) and *K. pneumoniae* (47%-55%) also observed with acidic ionized water. This finding is similar to an earlier report where alkaline electrolyzed water had no effect on *E. coli* growth [16]. Although, slightly acidic electrolyze water caused significant inhibition of *E. coli* growth [8,9], in this present study, acidic, neutral, and alkaline ionized water were inadequate to caused a bactericidal effect. The possible reason could be that ionized water when dissociated depending on whether it is acidic or alkaline, it only contains hydrogen ions, hydroxyl ions, and carbonate ions. It does not contain hypochlorous acid, which is one of the factors responsible for the antibacterial effect reported for acidic electrolyze water [17].

More so, *E. coli* and some other bacteria have been proved to possess the ability to maintain pH homeostasis [18,19]. Low pH environment favors generation of basic amines through the consumption of acid by enzyme dehydrogenases and decarboxylases with overall decrease in the acidic effect on the bacteria. While in high pH environment, there is upregulation of deaminases which results in cytoplasmic metabolic generation of ATP synthase, acid, and other important enzymes for the survival of the bacteria [20].

## Conclusion

This study revealed that acidic ionized water at pH 2.5 and 4.5 are effective in minimizing *Staphylococcus* spp. infection in horse wounds. The acidic ionized water could be of clinical importance as antiseptic for surface wound lavage. Therefore, future studies should aim to explain the antibacterial mechanism of ionized water on pathogenic bacteria for better understanding and institution of appropriate therapeutic processes.

## Authors’ Contributions

NMA and ZZ conceptualized and designed the study. AZAR and NMA collected the samples and AZAR performed the laboratory analysis. AMM did the data analysis. AZAR and AMM write the original draft. NMA and ZZ reviewed and edited the manuscript. All authors read and approved the final manuscript.
